# Porto-mesenteric vein thrombosis following laparoscopic sleeve gastrectomy for morbid obesity: Case series and literature review

**DOI:** 10.1016/j.ijscr.2019.09.004

**Published:** 2019-09-18

**Authors:** Naif A. Alenazi, Khaled S. Ahmad, Mohamed S. Essa, Mahir S. Alrushdan, Abdulbaset M. Al-Shoaibi

**Affiliations:** a Department of General Surgery, Prince Mohammed bin Abdulaziz Hospital, Riyadh, Saudi Arabia; b Department of General Surgery, Faculty of Medicine, Benha University, Benha, Egypt; c Department of Radiology, Prince Mohammed bin Abdulaziz Hospital, Riyadh, Saudi Arabia

**Keywords:** Porto-mesenteric vein thrombosis, Laparoscopic sleeve gastrectomy, Case series

## Abstract

**Background:**

Portal vein thrombosis is a rare and a potentially lethal complication of Laparoscopic sleeve gastrectomy. In this series, we describe the presentation, treatment, and outcome of 5 cases of PMVT post-laparoscopic sleeve gastrectomy (LSG) treated successfully at our hospital.

**Case report:**

Five patients presented to our emergency department with diffuse abdominal pain associated with anorexia, nausea and vomiting after laparoscopic sleeve gastrectomy (LSG). Computed tomography (CT) scan showed evidence of portal, mesenteric and splenic vein thrombosis and small bowel ischemia in three patients. Two patients were treated only with anticoagulant and the other three patients were treated with surgery in the form of diagnostic laparoscopy converted to laparotomy with Small bowel resection.

**Conclusion:**

Portal and mesenteric venous thrombosis after laparoscopic sleeve gastrectomy is an unusual complication but it has life threatening consequences if it is not diagnosed early and treated adequately. The patients with porto-mesenteric venous thrombosis (PMVT) usually present themselves with vague abdominal symptoms. As so, the physicians should have high index of suspicion to recommend computed tomography (CT) abdomen to confirm diagnosis and start adequate treatment.

## Highlights

• Porto-mesenteric vein thrombosis is rare and fatal complication after laparoscopic sleeve gastrectomy (LSG) for morbidly obese patients.
• Most patients presented with vague abdominal symptoms, so the physician should has high index of suspicion to recommend CT abdomen.
• Early diagnosis is of paramount importance for better outcome.
• Prophylactic anticoagulant is essential after LSG to prevent PMVT.

## 1 Introduction

The implementation of laparoscopic sleeve gastrectomy (LSG) for patients with morbid obesity is increasing [1]. It has been approved that LSG assists in weight loss as well as hindering comorbidities and improving the overall quality of life [1]. Porto-mesenteric vein thrombosis (PMVT) is a rare complication for laparoscopic sleeve gastrectomy (LSG); however, it could obviously impact the patients’ outcome and survival. Porto-mesenteric vein thrombosis (PMVT) occurs when the portal and mesenteric vein is occluded by thrombus either partially or entirely [2]. The thrombus may migrate to close portal vein branches, splenic veins, and mesenteric veins and the patients may present with various clinical manifestations such as fever and abdominal pain [3]. Herein, we aimed to report casesof portal vein, superior mesenteric vein, and splenic vein extensive thrombosis two weeks after undergoing laparoscopic sleeve gastrectomy LSG. The sleeve gastrectomies were performed in 3 different hospitals in the private sector prior to being referred to our hospital.

## 2 Case presentation

### 2.1 Case 1

A 43-year-old woman with a body mass index (BMI) of 37 kg/m^2^ presented at the emergency room complaining of sudden, severe, and generalized abdominal pain of one-day duration. The patient looked ill, dehydrated, and lethargic, confused, and had an altered level of consciousness as well as coffee ground vomiting. Local abdominal examination revealed generalized abdominal tenderness. Vital signs were disturbed; heart rate was 156 beats per minute, blood pressure was 81/58 mm Hg, respiratory rate was 26 breaths per minute, and body temperature was 36.5 °C.

**Fig. 1:**
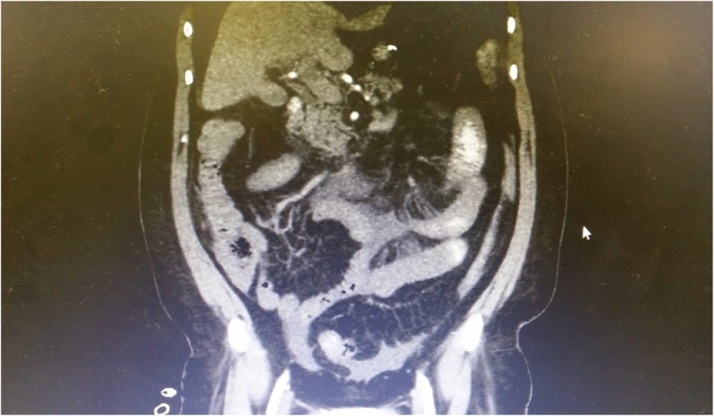
Computed tomography (CT) of abdomen showing porto-mesenteric vein thrombosis with evidence of small bowel ischemia.

Laboratory investigations were immediately performed and showed that the hemoglobin (Hgb) level was 13.4 g per deciliter, and white blood cell count (WBC) was 37,800 per microliter. Past history included laparoscopic sleeve gastrectomy for morbid obesity, two weeks prior to current admission with prophylactic anticoagulant for one week only after discharge. Computed tomography (CT) abdomen with intravenous (IV) contrast exhibited superior mesenteric vein, portal vein, and splenic vein thrombosis combined with signs of extensive small bowel ischemia (Fig. 1).

**Fig. 2:**
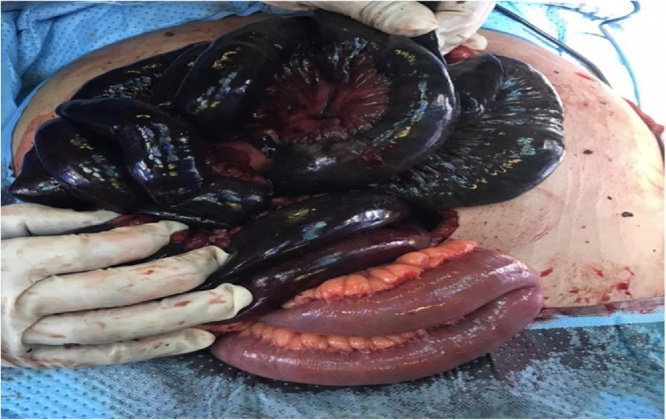
Open abdomen showing extensive small bowel ischemia.

Afterwards, diagnostic laparoscopy confirmed the findings, and hence exploratory laparotomy was carried out. The patient underwent small bowel resection saving 100 cm from ileocecal valve and 35 cm from Trietz ligament. The patient was left with an open abdomen for second look which indicated enduring small bowel ischemia (Fig. 2). Resection of 10 cm with side-to-side anastomosis was done during second look surgery with application of vacuum assisted closure device (VAC) for third look.

**Fig. 3:**
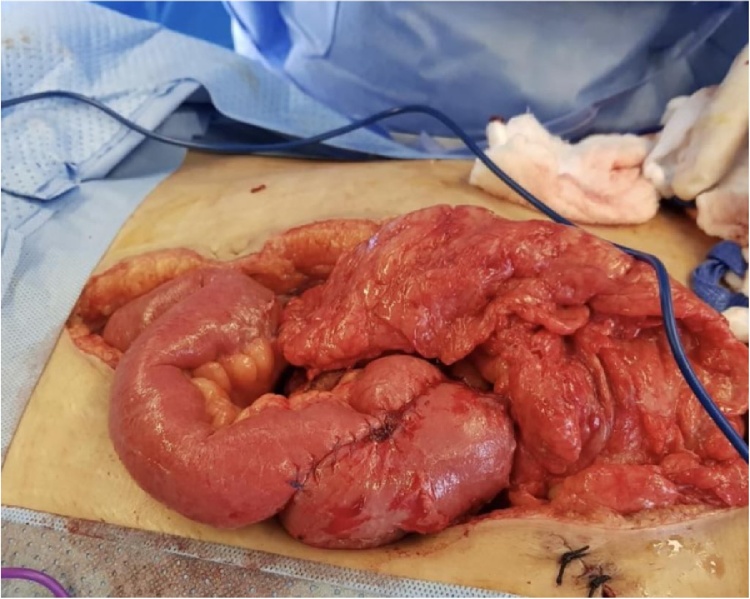
Open abdomen showing clean anastomotic abdominal field.

Third-time screening revealed clean anastomotic abdominal field and abdominal closure was straightway performed (Fig. 3). The patient was progressing well in the intensive care unit (ICU), extubated, and had well-tolerated nasogastric tube feeding. The patient received infusion with proton pump inhibitors (PPIs), tazobactam, and heparin.

On the ninth day, the patient experienced unexpected deterioration and computed tomography (CT) abdomen with IV contrast was ordered. The computed tomography (CT) displayed evidence of oral contrast leakage inside the peritoneal cavity, possibly at the site of the anastomosis. The physician decided abdominal re-exploration, which in turn, confirmed the computed tomography (CT) result. Catheter with inflatable balloon was placed in the site of the leak (anastomotic site) and total parenteral nutrition (TPN) was initiated.

After 48 h, examination showed no further leak, abdominal wash was done and vacuum-assisted closure (VAC) of the abdomen was applied. Two days later, computed tomography (CT) of abdomen with intravenous (IV) contrast was performed again and clarified clean abdomen. Abdominal closure was done and the patient was forwarded back to the primary physician after one week.

### 2.2 Case 2

A 22-year-old male patient with body mass index (BMI) of 38 kg/m^2^ came to the emergency department severely dehydrated with coffee ground vomiting, melena, and abdominal pain. The patient had history of laparoscopic sleeve gastrectomy (LSG) two weeks prior to present admission with only one week prophylactic anticoagulant after discharge. Vital signs were as follow; blood pressure was 151/66 mm Hg, respiratory rate was 24 breaths per minute, and body temperature was 36.6 °C. White blood cells (WBCs) level was 29,000, platelets count was 317,000, and prothrombin time was 2.0.Computed tomography (CT) of his abdomen indicated bowel ischemia secondary to porto-mesenteric thrombosis that has been confirmed through laparoscopic procedure.

**Fig. 4:**
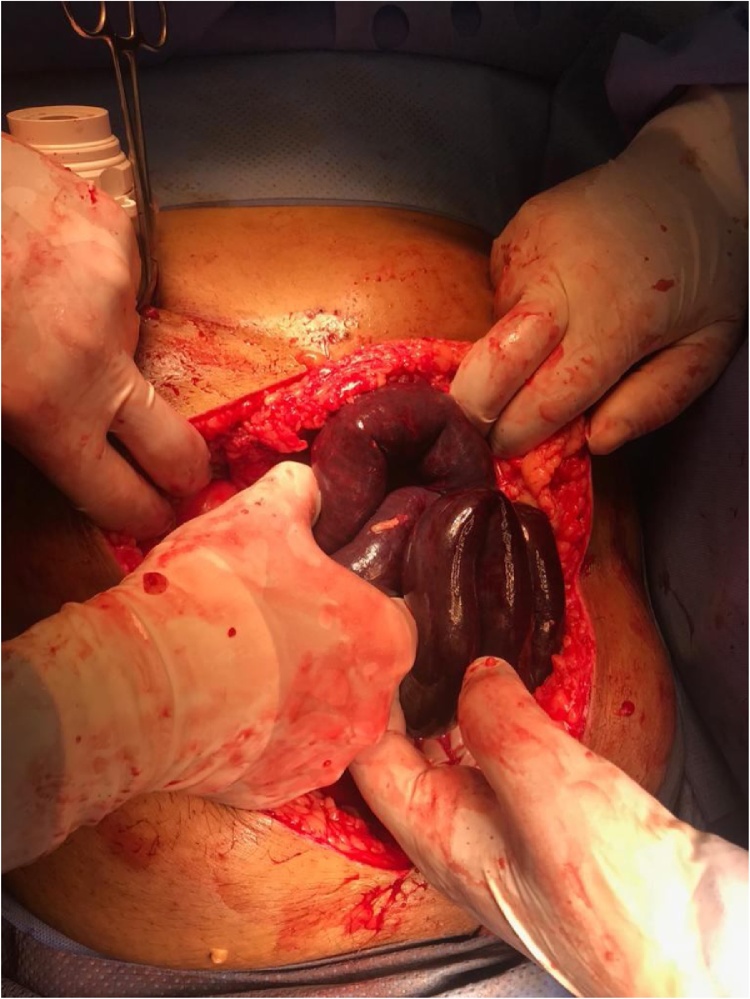
Open abdomen showing extensive small bowel necrosis.

Exploratory laparotomy was carried out and 80 cm from proximal jejunum was resected preserving 15 cm from duodeno-jejunal flexure with application of vaccum assisted closure (VAC) (Fig. 4). Patient started on total parenteral nutrition (TPN) and heparin infusion. After 48 h, examination showed no evidence of ischemia and hence hand sewn end-to-end anastomosis was done with abdominal wash and application of vaccum assisted closure (VAC). A third look, two days later, showed clean abdomen with no evidence of leak. Drains were inserted and the abdomen was closed. Five days later, the patient complained of melena; however, computed tomography (CT) abdomen with intravenous (IV) contrast showed no evidence of anastomotic leak. Also, upper endoscopy proved no evidence of active bleeding. The patient was discharged home on enoxaparin 80 mg twice daily and proton pump inhibitors (PPIs).

### 2.3 Case 3

**Fig. 5:**
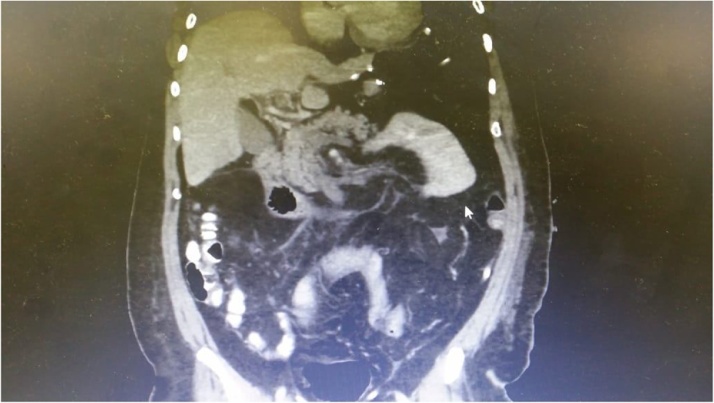
Computed tomography (CT) of the abdomen showing portal vein thrombosis without evidence of small bowel ischemia.

A 34-year-old female patient with a body mass index (BMI) of 49 kg/m^2^ was operated with laparoscopic sleeve gastrectomy (LSG). History included hypothyroidism treated by levothyroxine. Laparoscopic sleeve gastrectomy (LSG) was efficiently performed without any postoperative complications. On the second day, the patient was discharged home on heparin for one-week duration. One month after the surgery, the patient presented herself with constant and diffuse abdominal pain that progressed in severity over the following three days. Clinical examination revealed dehydrated patient with mild to moderate abdominal distention and tenderness. Interestingly, computed tomography (CT) of her abdomen showed extensive portal vein thrombosis with splenic vein thrombosis without evidence of bowel ischemia (Fig. 5). The patient was conservatively addressed with heparin and warfarin then discharged on follow-up with the primary physician.

### 2.4 Case 4

**Fig. 6:**
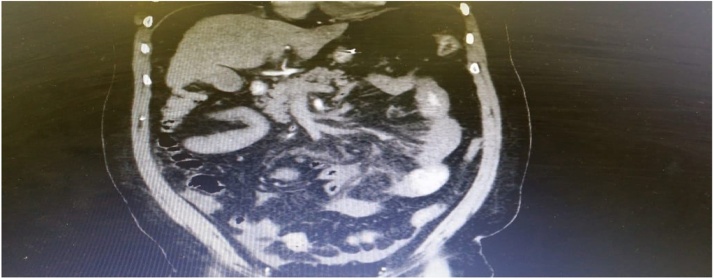
Computed tomography (CT) of the abdomen showed extensive portal, superior mesenteric vein and splenic vein thrombosis.

**Fig. 7:**
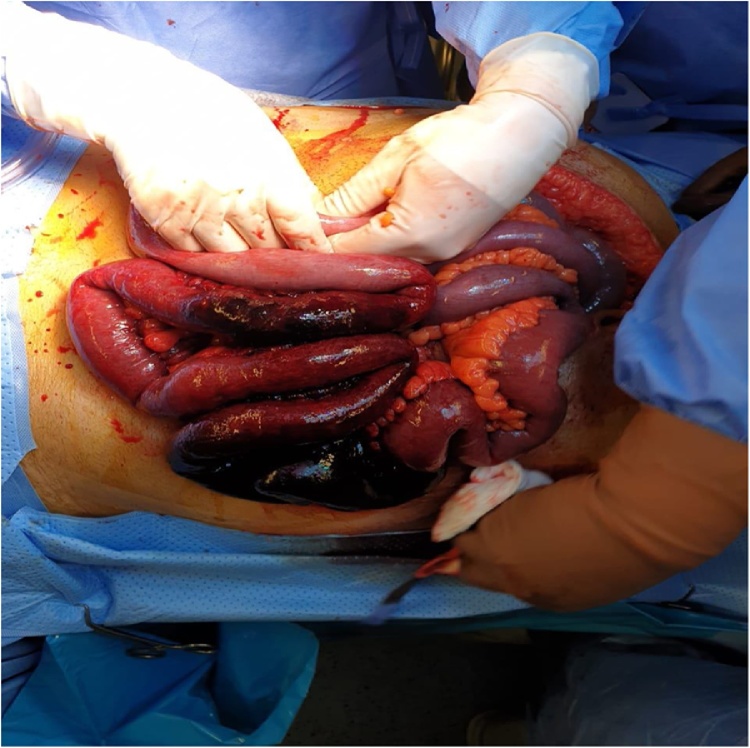
Open abdomen showing extensive small bowel ischemia.

A 53-year-old male patient with body mass index (BMI) of 42 kg/m^2^ came to the emergency department dehydrated with diffuse abdominal pain and vomiting. The patient had history of laparoscopic sleeve gastrectomy (LSG) two weeks prior to present admission with only one week of venous thrombo-embolic prophylaxis. Vital signs were as follow; blood pressure was 140/60 mm Hg, respiratory rate was 25 breaths per minute, and body temperature was 37.4 °C. White blood cells (WBC) level was 25,000, platelets count was 310,000, and prothrombin time was 2.0. The CT of his abdomen showed extensive portal vein thrombosis involving portal vein, superior mesenteric vein and splenic vein with evidence of small bowel ischemia affecting jejunum (Fig. 6). The diagnosis has been confirmed through diagnostic laparoscopy. Exploratory laparotomy was carried out and 85 cm from the proximal jejunum resected, vacuum assisted closure (VAC) was applied and the patient shifted to intensive care unit (ICU) (Fig. 7). Patient started on total parental nutrition TPN and heparin infusion. After 48 h, second look surgery showed another ischemic segment about 10 cm was resected with hand sewn end –to-end anastomosis done, vaccum assisted closure (VAC) machine was applied. After 48 h, a third look surgery was done and showed no evidence of leak and ischemia. And so, the abdomen was closed over drains. Postoperative course was uneventful and the patient was discharged home on enoxaparin 80 mg twice daily and proton pump inhibitors (PPIs).

### 2.5 Case 5

A 25-year-old male patient with a body mass index (BMI) of 50 kg/m^2^, admitted for laparoscopic sleeve gastrectomy (LSG). On the second day, the patient was discharged home without any prophylactic anticoagulant. Two weeks after the surgery, the patients presented with dehydration and diffuse abdominal pain that progressed in severity over the last two days. Vital signs were as follow; blood pressure was 130/70 mm Hg, respiratory rate was 22 breaths per minute, and body temperature was 36.7 °C. Abdominal examination showed soft lax abdomen without tenderness. White blood cells (WBC) level was 11000, platelets count was 310,000, and prothrombin time was 1.6. Interestingly, computed tomography (CT) of his abdomen showed extensive porto-mesenteric venous thrombosis without evidence of bowel ischemia. The patient was conservatively treated with heparin and warfarin then discharged on follow-up with the primary physician.

Our case series has been reported in line with the PROCESS criteria

## 3 Discussion

The mounting applications of laparoscopic sleeve gastrectomy (LSG) for patients with morbid obesity urged healthcare professionals to investigate post-operative complications. Of note, deep venous thrombosis, pulmonary embolism, hemorrhage, and anastomotic leakage are commonly reported complications in the post-operative course of laparoscopic sleeve gastrectomy (LSG) [1,4]. Porto-mesenteric vein thrombosis (PMVT) is a potentially fatal complication of LSG; however, this has been rarely reported in the literature [5,6]. This series discusses cases that were referred to our hospital with porto-mesenteric venous thrombosis (PMVT) for patients who had a past history of laparoscopic sleeve gastrectomy (LSG) for morbid obesity in 3 different hospitals, two weeks prior to being admitted in our emergency room.

Although the porto-mesenteric venous thrombosis (PMVT) is a lethal complication in the postoperative course of laparoscopic sleeve gastrectomy(LSG), the diagnosis is very deceptive, owing to the nonspecific complaints. Patients may experience abdominal discomfort or pain, fever, nausea or vomiting. Nevertheless, some porto-mesenteric venous thrombosis (PMVT) cases could be asymptomatic and discovered accidentally through radiological examination [3]. The presented cases had severe leukocytosis and disturbed vital signs, physicians might also encounter mild rising in liver function tests; however, laboratory values fall usually within normal limits [6]. The diagnosis of porto-mesenteric venous thrombosis (PMVT) is typically performed through contrast computed tomography (CT) scan and Doppler ultrasound; our patients had only contrast computed tomography (CT) that showed porto-mesenteric venous thrombosis (PMVT), as well as small bowel ischemia.

It is worth mentioning that porto-mesenteric venous thrombosis (PMVT) not only occurs after laparoscopic sleeve gastrectomy (LSG) but also has been reported after different laparoscopic surgical proceedings like splenectomy, cholecystectomy, and fundoplication [7,8]. Nevertheless, the exact etiology has not yet clearly been understood. The experimental studies deduced that the intra-abdominal pressure produced during laparoscopic surgery has an inverse relation with the blood flow within the portal vein, which in turn, prepare for thrombus formation [9]. The release of vasopressin during operation, reverse Trendelenburg posture applied during the laparoscopic intervention, and the elevation of portal venous pressure in response to retained carbon dioxide (Co_2_) may also be contributing factors [9]. In our cases the main risk factors are dehydration and improper duration of prophylactic anticoagulant.

The advantages of prohylactic anticoagulation given routinely after bariatric surgery is accepted to a large degree. Both low molecular weight heparin (LMWH) and unfractionated heparin (UFH) can be used in bariatric surgery; however, until now, there is no strong evidence regarding dose and duration of prophylactic heparin [10]. However, it is noted that there is no data demonstrating relation between deep vein thrombosis (DVT) and regarding pathophysiology, prevention, management, and complications; and so, deep venous thrombosis (DVT) prophylaxis given for surgical patients can be used in porto-mesenteric venous thrombosis (PMVT).

One study demonstrated that duration of 10 days of prophylactic heparin after bariatric surgery is effective [11]. Other studies noted that prophylactic enoxaparin given preopertaively at the time of surgery was associated with significant rate of major bleeding without reduction in deep venous thrombosis (DVT) incidence. It appeared that 40 mg of enoxaparin given two times daily is superior to 30 mg twice daily and after discharge administration of enoxaparine 40 or 50 mg once a day is efficient [12].

The guidelines of the American Association of Clinical Endocrinologists (2013), the American Society for Metabolic and Bariatric Surgery Medical Guidelines for Clinical Practice for the Perioperative Nutritional, Metabolic, and Nonsurgical Support of the Bariatric Surgery Patient and obesity society(AACE/ASMBS/TOS guidelines) recommended that regimens of prophylactic heparin following bariatric surgery include intermittent pneumatic compression devices in addition to subcutenous injection of low molecular weight heparin (LMWH) and unfractionated heparin (UFH). Deep venous thrombosis (DVT) prophylaxis for long period (extended prophylaxis) should be given for patients with high risk factors such as patients with past history of deep venous thrombosis (DVT) [13].

One of the methods for deep venous thrombosis (DVT) risk stratifictaion is modified Caprini score [14].With application of this score on our reported patients, most of them were at high risk.

In comparison to Roux-en-Y (RYGB), porto-mesenteric venous thrombosis (PMVT) is rare and has fatal consequences, but the recorded cases are less than reported after laparoscopic sleeve gastrectomy (LSG). Inspite of both types of surgery having the same riskfactors, presentation, onset of diagnosis after surgery and management results, there is no elucidation for this contradiction [15].

There are several treatment options for porto-mesenteric venous thrombosis (PMVT) based on the degree and severity of thrombosis (occlusive versus non occlusive) which includes oral anticoagulant, thrombolytic therapy and thrombectomy using tissue plasminogen activator (TPA) [16]. 60% of patients receiving thrombolytic therapy developed major complications [17].

If there is evidence of ischemic bowel, laparotomy is better than laparoscopy because of insufflation of the abdominal cavity with gas which increases the pressure inside mesenteric veins (exaggerate venous hypertension) in addition to extensive bowel edema and abdominal distenveins that make laparoscopic access difficult [18]. In our case, the extensive small bowel ischemia urged the exploratory laparotomy and intestinal resection of necrotic parts as an emergency solution.

Standard anti-coagulation therapy is usually applied for patients with portal vein thrombosis (PVT) and can entirely lead to thrombus resolution in mild to moderate cases [19]. There are retrospective studies reported that patients with acute portal vein thrombosis treated with anticoagulation, partial recanalization of the portal vein occurred in 63%–93% of patients and complete recanalization occurred in 34%–45% [[20], [21], [22], [23], [24]].

## 4 Conclusion

To recapitulate, laparoscopic sleeve gastrectomy (LSG) for morbid obesity is not a complication free procedure. Patients undergoing laparoscopic sleeve gastrectomy (LSG) might experience porto-mesenteric venous thrombosis (PVT) a few weeks after the operation. The presenting clinical features are vague; abdominal pain, vomiting, abdominal discomfort, fever, and nausea. Therefore, the healthcare professionals are encouraged to consider porto-mesenteric venous thrombosis (PMVT) in the differential diagnosis. Future studies are recommended to construct rigorous and standard treatment and prophylaxis procedures.

## Funding

There are no sources of funding to acknowledge.

## Ethical approval

Our study exempted from ethical approval.

## Consent

Written informed consent was obtained from the patient for publication of this case series and any accompanying images.

## Author’s contribution

**Naif A. Alenazi**; **Khaled S. Ahmed**; **Mohamed S. Essa**; **Mahir S Alrushdan** and **Abdulbaset M. Al-Shoaibi** – Conception of the work, Design of the work, Drafting the work, Revising the work critically for important intellectual content, Final approval of the version to be published, Agree to be accountable for all aspects of the work in ensuring that questions related to the accuracy or integrity of any part of the work are appropriately investigated and resolved.

## Registration of research studies

Our study under review stage in Clinical Trial Registry-India (CTRI).Reference number is REF/2019/05/026144.

## Guarantor

The corresponding author is the guarantor of submission.

## Provenance and peer review

Not commissioned, externally peer-reviewed.

## Declaration of Competing Interest

There is no conflict of interest to disclose.
